# lncRNA *Spehd* Regulates Hematopoietic Stem and Progenitor Cells and Is Required for Multilineage Differentiation

**DOI:** 10.1016/j.celrep.2019.03.080

**Published:** 2019-04-16

**Authors:** M. Joaquina Delás, Benjamin T. Jackson, Tatjana Kovacevic, Silvia Vangelisti, Ester Munera Maravilla, Sophia A. Wild, Eva Maria Stork, Nicolas Erard, Simon R.V. Knott, Gregory J. Hannon

**Affiliations:** 1 Cancer Research UK Cambridge Institute, Li Ka Shing Centre, University of Cambridge, Cambridge CB2 0RE, UK; 2 Watson School of Biological Sciences, Howard Hughes Medical Institute, Cold Spring Harbor Laboratory, Cold Spring Harbor, NY 11724, USA; 3 New York Genome Center, New York, NY 10013, USA

**Keywords:** lncRNA, hematopoiesis, HSC, oxidative phosphorylation

## Abstract

Long non-coding RNAs (lncRNAs) show patterns of tissue- and cell type-specific expression that are very similar to those of protein coding genes and consequently have the potential to control stem and progenitor cell fate decisions along a differentiation trajectory. To understand the roles that lncRNAs may play in hematopoiesis, we selected a subset of mouse lncRNAs with potentially relevant expression patterns and refined our candidate list using evidence of conserved expression in human blood lineages. For each candidate, we assessed its possible role in hematopoietic differentiation *in vivo* using competitive transplantation. Our studies identified two lncRNAs that were required for hematopoiesis. One of these, *Spehd*, showed defective multilineage differentiation, and its silencing yielded common myeloid progenitors that are deficient in their oxidative phosphorylation pathway. This effort not only suggests that lncRNAs can contribute to differentiation decisions during hematopoiesis but also provides a path toward the identification of functional lncRNAs in other differentiation hierarchies.

## Highlights

• Conserved differential expression of syntelogs to enrich functional lncRNAs
• Bone marrow transplant of lncRNA-depleted HSCs uncovers *in vivo* functional lncRNAs
• *Spehd* is required for differentiation during reconstitution and homeostasis
• Myeloid progenitors depleted of *Spehd* show oxidative phosphorylation defects

## Graphical Abstract

**Figure:**
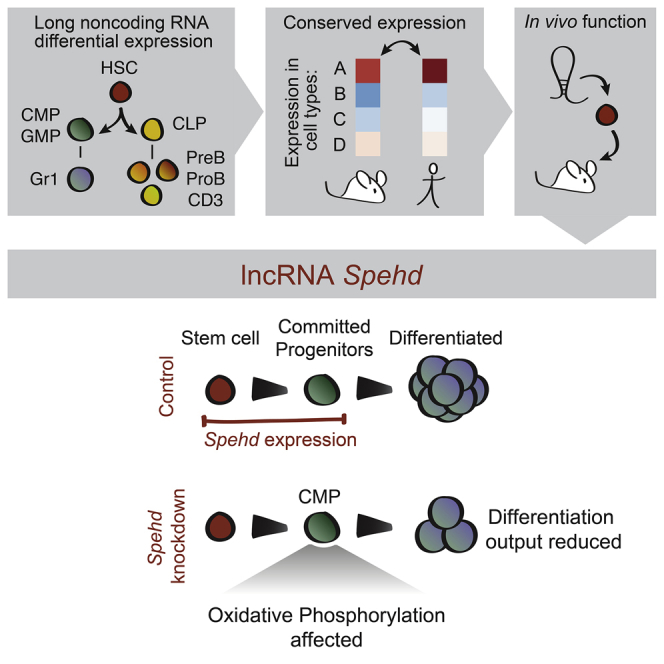

## Teaser

Delás et al. identify *Spehd* as an lncRNA expressed in hematopoietic stem and progenitor cells and required for *in vivo* differentiation during transplantation and homeostasis.

## Introduction

Long noncoding RNAs (lncRNAs) can function as regulators of cell fate via a number of mechanisms, ranging from gene expression regulation to effects on mRNA and protein stability (Delás and Hannon, 2017). While there are many thousands of lncRNAs annotated in different genomes (Melé and Rinn, 2016), the challenge of identifying those that regulate a particular process is still significant. With the breadth of information that the field has produced on hematopoietic stem cell (HSC) differentiation, the hematopoietic system is the perfect model to investigate how lncRNAs regulate differentiation (Orkin and Zon, 2008). The power of this system hinges on the ability to perform *in vivo* bone marrow reconstitutions, the ultimate proof of biological relevance.

Regulation of cell fate transitions during hematopoiesis has been studied at many different levels. Transcription factors essential for various steps of hematopoietic differentiation (Orkin and Zon, 2008) are often positive regulators of their own transcription, forming a highly dynamic transcription factor network (Schütte et al., 2016). This tight regulation of gene expression is also highly dependent on additional transcriptional control mechanisms, such as DNA methylation changes (Challen et al., 2011, Challen et al., 2014, Trowbridge et al., 2009) and chromatin modifications (Kerenyi et al., 2013). Post-transcriptional regulation through microRNAs has also been described, with the most notable examples being *MicroRNA-126*, which promotes HSC quiescence (Lechman et al., 2012), and miR-223, required for granulocyte differentiation (Fukao et al., 2007, Johnnidis et al., 2008).

The function of several lncRNAs has been addressed in *in vitro* models of hematopoietic differentiation, such as granulocyte differentiation (Zhang et al., 2009), eosinophil differentiation (Wagner et al., 2007), and erythropoiesis (Hu et al., 2011). Global analysis of annotated lncRNAs has also revealed that their expression is regulated in early stem cell populations (Cabezas-Wallscheid et al., 2014). Since the current GENCODE annotation for lncRNAs is mostly based on easy-to-culture cell lines or whole organisms, it lacks many of the cell type-specific hematopoietic transcripts. To circumvent this, some groups have assembled annotations for subsets of the hematopoietic lineage or for some of the differentiation models mentioned above (Alvarez-Dominguez et al., 2014, Luo et al., 2015, Paralkar et al., 2014). We recently sought to produce a robust annotation that encompassed cell types from HSCs to differentiated cells, both myeloid and lymphoid lineages, as well as blood cancers (Delás et al., 2017). In our first proof-of-concept study, we used this resource to characterize lncRNAs required for acute myeloid leukemia (AML).

Here, we focused on characterizing lncRNAs involved in the earliest choices the HSC must make: self-renewal or commitment to a lineage. To address this question, we devised an experimental strategy whereby long-term reconstituting HSCs could be transduced *in vitro* with short hairpin RNAs (shRNAs) targeting lncRNAs and then transplanted to uncover lncRNA dependencies *in vivo*. Since many aspects of hematopoiesis are conserved between mice and humans (Shay et al., 2013), we reasoned that if we could identify lncRNAs with syntenic conservation and conserved expression in humans, then we could enrich for functional potential.

## Results

### Combination of Differential Expression with Syntenic Conservation and Conserved Expression to Enrich Functional lncRNAs

**Figure 1:**
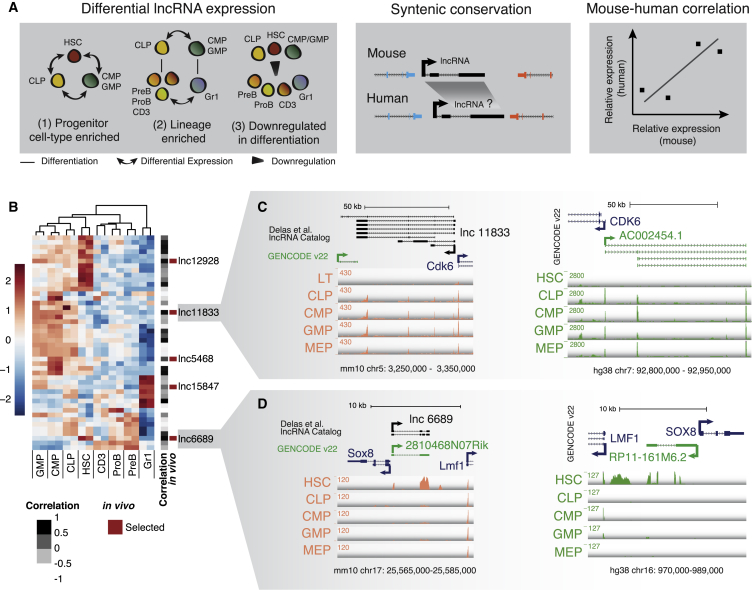
An Approach to Enrich Potentially Functional lncRNAs (A) Pipeline overview from the expression patterns selected to mouse-human expression correlation. (B) Expression heatmap of the 45 mouse lncRNAs that have expressed syntelogs in humans, indicating their level of expression correlation and the lncRNAs selected for *in vivo* studies. (C and D) Genome browser plots for the mouse (left) and human (right) loci for lnc11833-AC002454.1 (C) and lnc6689/2810468N07Rik-RP11-161M6.2 (D). See also Figure S1 and Table S1.

We hypothesized that if the expression of a candidate lncRNA was tightly regulated during hematopoietic differentiation, then it would be more likely to be involved in regulating cell fate transitions than would a ubiquitously expressed transcript. To maximize the likelihood of identifying lncRNAs functionally required for HSC differentiation and/or self-renewal, we used expression analysis and combined lncRNA annotations with (1) enriched expression in HSCs, committed myeloid or granulocyte-monocyte (CMP/GMP), or lymphoid progenitors (CLP) when we analyzed differential expression between those cell types (progenitor cell type enriched); (2) differential expression between myeloid and lymphoid lineages that already displayed primed differential expression at the progenitor level (lineage enriched); and (3) differential downregulation during differentiation while already differentially expressed between any of the progenitor or stem populations (downregulated in differentiation) (Figure 1A). This generated a list of 295 mouse lncRNAs with suggestive expression patterns (Figure S1A).

lncRNAs are often not conserved in sequence, but one can often find an lncRNA in a syntenic position in different genomes (Hezroni et al., 2015). We found that in 97 cases, we could identify an annotated lncRNA in a syntenic position in the human genome. We then selected lncRNAs expressed in human cord blood HSC, CMP, GMP, and CLP (Chen et al., 2014), yielding a list of 45 lncRNAs.

We analyzed the expression correlation for each of these lncRNAs in humans and mice for the aforementioned cell types to prioritize lncRNAs for *in vivo* studies. Due to limitations in lncRNA assemblies and the complex genomic organization of some loci, we assembled our final list of five lncRNAs after manual inspection of each genomic locus to ensure that the human expression data were reflective of the lncRNA levels (and not an overlapping gene) and that the lncRNAs selected showed a consistent exon structure across replicates (Figures 1B–1D and S1B–S1D; Table S1).

*lnc11833*, for example, is divergently transcribed with *Cdk6* and dramatically upregulated in all committed progenitors as compared to stem cells in both mice and humans (Figure 1C). Given the reported role of *Cdk6* in regulating human HSC quiescence (Laurenti et al., 2015), the location and expression of this lncRNA was potentially suggestive of an effect in *cis*. Another lncRNA, *lnc6689*, is expressed in HSCs but not in the progenitor cell types (Figure 1D). While *lnc6689* (*2810468N07Rik* in GENCODE) is also expressed divergently from a neighboring protein-coding gene, *Sox8*, this gene is not expressed in blood progenitors and does not have any known role in hematopoiesis.

We also identified an already described lncRNA that overlaps with *miR-223* (*lnc15847*, *F630028O10Rik*) (Figure S1B). This microRNA plays a role in myelopoiesis (Fazi et al., 2007, Fukao et al., 2007), and the lncRNA has recently been implicated in AML (Mangiavacchi et al., 2016).

### Bone Marrow Transplantation Identifies lncRNAs Required for Hematopoiesis *In Vivo*

**Figure 2:**
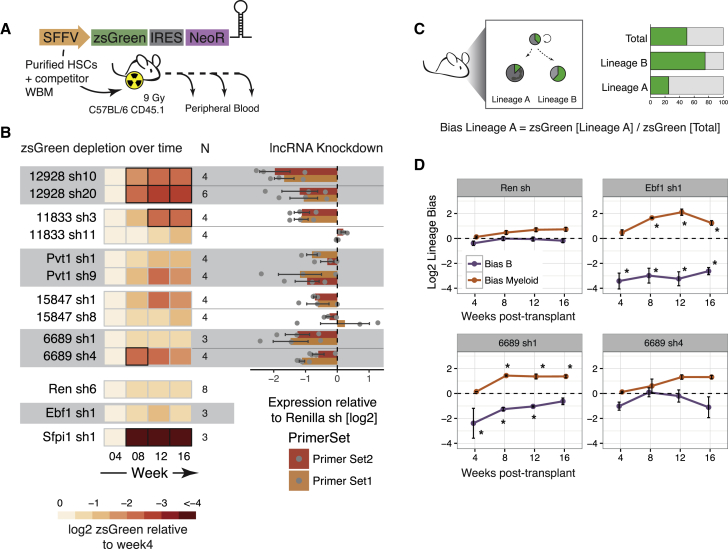
*In Vivo* Reconstitution with lncRNA-Depleted HSCs Identifies lncRNAs Involved in Overall Differentiation or Lineage Specification (A) Schematic representation of the vector used and the experimental design. NeoR, neomycin resistance gene. WBM, whole bone marrow. (B) Heatmap depicting the average depletion of zsGreen^+^ cells relative to the week 4 measurement (left) and the corresponding level of knockdown in a cell line that expresses the corresponding lncRNA (see STAR Methods) for each shRNA assayed *in vivo* (right). N is the number of mice analyzed for each knockdown. Black boxes represent significantly depleted time points (p < 0.05; Mann-Whitney test). (C) Schematic representation of the concept of lineage bias analysis. (D) Average lineage bias for the myeloid and B lineages at the different time points in the blood for the indicated knockdowns. The error bars represent SEMs. The raw data from this analysis are the same as in (B). ^∗^p < 0.05; Mann-Whitney test. See also Figure S2.

To assess the functional importance of our selected lncRNAs *in vivo*, we performed bone marrow reconstitutions, transplanting HSCs transduced with an shRNA against the lncRNAs. shRNAs were designed with our previously published algorithm for the prediction of highly potent shRNAs (Knott et al., 2014) and cloned into a constitutive lentiviral vector where a spleen focus-forming virus (SFFV) promoter drives the expression of a green fluorescent protein (zsGreen) and an shRNA. Sorted CD45.2 E-SLAM (CD45^+^/EPCR^+^/CD48^–^/CD150^+^) HSCs (Kent et al., 2009) were transduced with high titer lentivirus for ∼20 h before injecting them into irradiated recipient CD45.1 animals. The cells were mixed with competitor CD45.1 whole bone marrow before injection. Peripheral blood was analyzed starting at 4 weeks post-transplant (Figures 2A and S2A). Because only a fraction of HSCs transplanted had been transduced, we monitored the zsGreen percentage within the donor compartment over time as a phenotypic readout. If the shRNA and zsGreen-expressing cells were less able to repopulate the hematopoietic compartment as compared to the non-transduced counterparts, then we would expect a depletion of zsGreen^+^ cells over time.

We saw a dramatic decrease in the representation of zsGreen^+^ cells transduced with an shRNA against positive control *Spi1* (also known as *PU1*) as compared to cells expressing an shRNA against our negative control, *Renilla* luciferase (Figure 2B). *Spi1* is a transcriptional activator that is indispensable for HSC self-renewal and commitment to and maturation of myeloid and lymphoid lineages (Iwasaki et al., 2005). We therefore would expect almost no output of differentiated cells from HSCs in which *Spi1* was silenced. Of note, because we do not have a robust way of measuring initial HSC infection rates (many animals would be required to measure the initial infection of this very rare population), we normalize to the initial blood measurement at 4 weeks post-transplant. By that point, the percentage of zsGreen-expressing cells could already be greatly reduced if the targeted RNA were required for HSC differentiation or maintenance.

Using this readout, we identified *lnc12928* as our strongest candidate. Using two independent shRNAs, we saw between 4- and 8-fold average reduction in the relative number of zsGreen^+^ cells within the donor compartment during the course of the experiment (Figures 2B and S2B). To verify that we were affecting the expression of the targeted lncRNAs with our shRNAs, we performed a knockdown analysis on cell lines that express each of the targeted lncRNAs. Using this approach, we confirmed that the levels of *lnc12928* were greatly reduced using both shRNAs and when measured with two independent primer pairs (Figure 2B).

We noticed that the only shRNA that successfully silenced *lnc11833* was also the only one that gave rise to a depletion of zsGreen-expressing cells *in vivo* (Figures 2B and S2B). This suggests that *lnc11833* could still be an important lncRNA in hematopoiesis, although additional lncRNA depletion experiments with other tools would be required to validate this observation.

These results show that our HSC transduction followed by transplantation approach is a useful way to identify the genes that are required for hematopoietic reconstitution in general, and is a powerful tool to identify functional lncRNAs.

### HSCs Depleted of *lnc6689* Display a Lineage-Bias Phenotype

lncRNAs could affect hematopoiesis in a variety of ways that would not necessarily lead to changes in the overall representation of zsGreen^+^ cells. For example, our lineage control in this assay is early B cell factor 1 (*Ebf1*), a gene required for B cell differentiation (Zandi et al., 2008). *Ebf1* knockdown does not result in changes in the relative fraction of zsGreen^+^ cells, as compared to week 4 post-transplant (Figure 2B). However, we observed that within each animal, the percentage of zsGreen^+^ cells in the B220^+^ compartment was much lower than in the myeloid cell types (Figure S2C). To investigate this further, we assessed the percentage of zsGreen^+^ cells within the B cell donor compartment relative to the overall zsGreen^+^ population derived from the donor (lineage bias), and performed the same analysis for the myeloid lineage (Figures 2C, S2B, and S2C). This showed a clear bias against the B lineage and enrichment of the myeloid compartment for *Ebf1* knockdown (Figure 2D). *Renilla* shRNAs, in contrast, showed a generally balanced phenotype, where the fraction of zsGreen^+^ cells within each lineage mirrored the representation of zsGreen^+^ populations in blood overall.

By performing the same lineage-bias analysis, we found that one of our lncRNAs, *lnc6689*, had a potential bias against B cells or in favor of the myeloid lineage (Figures 2D and S2C). We see this phenotype with both shRNAs targeting this lncRNA, although the extent of the effect differs. This indicated that each zsGreen-expressing HSC transplanted is producing fewer B220^+^ progeny or more myeloid cells. The extent of this phenotype correlated with the degree of knockdown we observed when this lncRNA was targeted in A20 cells (Figures 2B and S2B), suggesting a dose-dependent effect.

While *lnc6689* has striking HSC-specific expression among progenitor cell types (Figure 1D), we also observed its presence in pre- and pro-B cells (Figure S3A), which means that the lineage-biased phenotype could be mediated either by its functioning in HSCs or via an effect during B cell differentiation.

**Figure 3:**
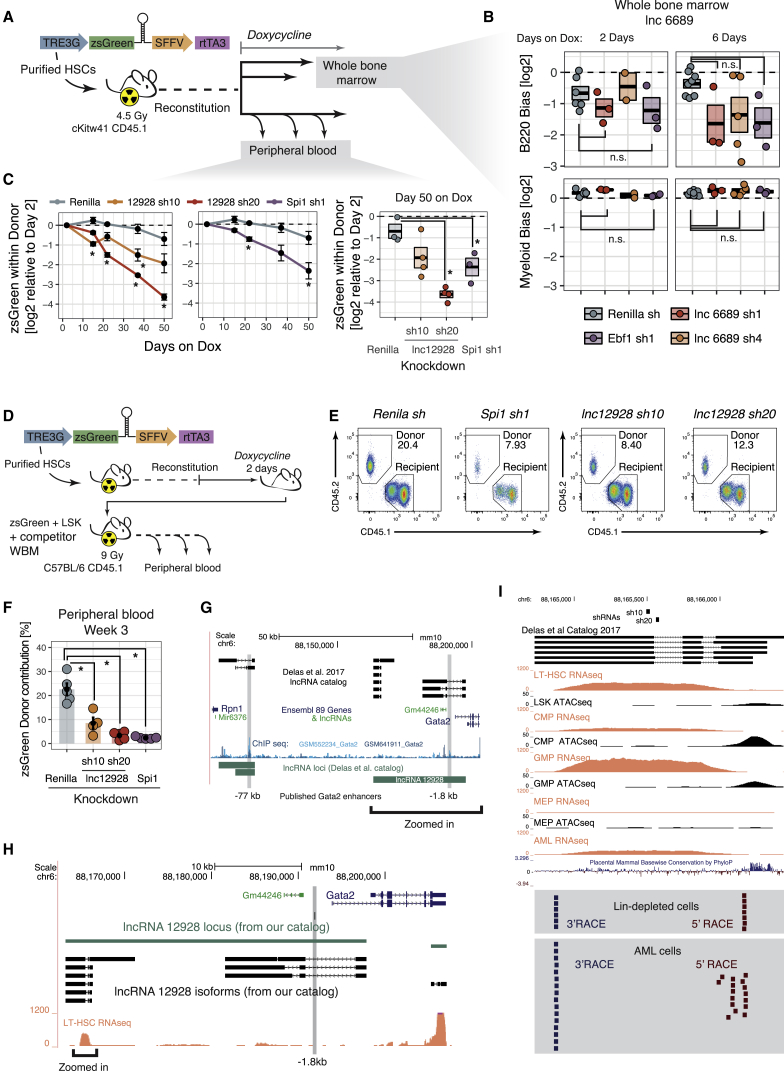
lnc6689 Knockdown Results in B Cell Depletion and Myeloid Enrichment, while lnc12928 Is Required for Multilineage Differentiation (A) Schematic representation of the vector used and the experimental design for bone marrow transplants using inducible shRNAs. (B) Lineage-bias values for the B and myeloid lineages in the bone marrow for the knockdowns indicated in animals induced with doxycycline for 2 or 6 days. The center of the box indicates average and the box edges represent SEM. (C) Proportion of zsGreen^+^ cells within donor peripheral blood relative to 2 days post-administration of doxycycline for each shRNA over time (left [days on dox], average and SEM presented for each time point). For the last time point (50 days), the values for each animal are represented (right). The center of the box indicates average and the box edges represent SEM. ^∗^p < 0.05; Welch’s unequal variances t test. (D) Schematic representation of the vector used and the experimental design for re-transplantations. (E) Representative flow cytometry plots of the peripheral blood of animals transplanted solely with zsGreen^+^ LSK, as described in (D), for each of the conditions analyzed. (F) zsGreen donor percentage for each re-transplanted animal at 3 weeks post-transplant. The bar heights indicate the average zsGreen percentage per condition; the error bars represent SEMs. ^∗^p < 0.05; Welch’s unequal variances t test. (G) Genome browser representation of the predicted *lnc12928* locus and its neighboring genes *Gata2* and *Rpn1*. The location of the published *Gata2* enhancers is indicated. *Gata2* chromatin immunoprecipitation sequencing (ChIP-seq) tracks are displayed as obtained from codex.stemcells.cam.ac.uk. (H) Magnified region as indicated in (G), showing the RNA-seq coverage for long-term HSC (LT-HSC). (I) Magnified region as indicated in (H), showing the transcript starts and ends, as defined by 5′/3′ RACE. The RNA-seq coverage is presented for the cell types indicated. The assay for transposase accessible chromatin with high-throughput sequencing (ATAC-seq) came from Lara-Astiaso et al. (2014). The location of the shRNAs used against this lncRNA are also presented. Lin, lineage. See also Figure S3.

To establish a strategy in which we could address potential cell type-specific effects, we built an inducible shRNA vector that would allow us to perform bone marrow reconstitutions and only activate shRNA expression once the hematopoietic system has been repopulated. With this approach we could acutely induce an shRNA and investigate its impact in different cell types or at different time points (Figure 3).

We transduced cells with inducible shRNAs against *lnc6689* or controls, *Renilla* and *Ebf1,* and transplanted them into cKit(w41);CD45.1, a mouse strain that is particularly suitable as an HSC recipient and that only requires sublethal irradiation. We allowed the animals to reconstitute and induced the shRNA by feeding doxycycline-containing food (Figure 3A). When we looked at the whole bone marrow (after lysing red blood cells) of these animals, we saw a bias against the B220^+^ compartment for cells in which *Ebf1*, our lineage control, was silenced, with no change in the myeloid compartment, which constitutes the large majority of the non-progenitor cells in the bone marrow. The extent of the bias increased in magnitude if we administered doxycycline to the animals for 6 days rather than 2 days (Figure 3B), but it is not statistically significant in either case. Although we also see a bias against the B220^+^ compartment in the *lnc6689* knockdown, the phenotype is less severe and quite variable, especially with shRNA 4 (Figure 3B). Additional experimental efforts with different depletion tools and at later time points will be required to further validate this phenotype and the role of *lnc6689* in either stem cells or the B cell compartment directly. For these reasons, we decided to focus our attention on *lnc12928*.

### *lnc12928 (Spehd)* Is Required for Hematopoiesis during Regeneration and in Homeostasis Post-transplantation

Using the same inducible system described above, we sought to investigate whether the *lnc12928* requirement we noted in animals during reconstitution was also observed if the knockdown was induced after the hematopoietic system had recovered following transplantation. We found that the representation of zsGreen^+^ cells within the donor compartment decreased over time in the peripheral blood of animals transplanted with HSCs with *lnc12928* knockdown, compared to the initial percentage 2 days post-administration of doxycycline (Figures 3C, S3B, and S3C). For shRNA 20, the effect of the depletion is similar to that of the positive control knockdown, *Spi1* (*PU1*), which is known to be essential in HSCs (Figure 3C).

This confirms that *lnc12928* is required for hematopoiesis, both in the setting of reconstitution and for homeostasis post-transplant. This lncRNA is highly enriched in hematopoietic progenitors, but not in any of the differentiated cell types (Figure S3A). Consequently, these data suggest that this lncRNA must exert an effect at the level of the stem and/or progenitor cell types. We therefore named this lncRNA *Spehd* (stem and progenitor enriched required for hematopoietic differentiation).

The predicted “lncRNA locus” (i.e., the collection of all of the possible isoforms) in our catalog extended upstream of the area where we observed strong RNA sequencing (RNA-seq) coverage in our cell types to the start of its upstream gene, *Gata2* (Figure S1). This predicted locus would overlap with a known *Gata2* enhancer at −1.8 kb (Snow et al., 2010) (Figures 3G and 3H). To better characterize this lncRNA, we performed 3′ and 5′ rapid amplification of cDNA ends (RACE) in AML cells, which was further validated with lineage-depleted bone marrow cells. This confirmed that the lncRNA start and termination sites flanked the area of high RNA-seq coverage, where our shRNAs were designed (Figure 3I). Previously published DNA accessibility data (Lara-Astiaso et al., 2014) further support start site location. Regarding the structure of the lncRNA, only 3 of 21 cDNA Sanger sequencing reads that would span the predicted first intron and 0 of 43 that covered the second showed evidence of splicing (data not shown). This would suggest that the predominant isoform is not spliced, which would be in line with the RNA-seq coverage, although a much larger number of reads would be required to confidently estimate isoform abundances. These RACE data also exclude any genomic overlap between *Spehd* and another previously described *Gata2* enhancer at −77 kb (Grass et al., 2006) (Figure 3G).

### Depletion of *Spehd* Results in Impaired Stem and/or Progenitor Contribution to Hematopoiesis

We next isolated shRNA-expressing cells that we could transplant into recipient animals as the sole source of donor cells. Since we expected that for some of the knockdowns these cells would have a deleterious reconstitution phenotype, we used the same competitive transplantation setup as our initial studies (Figure 2A), using C57BL/6-CD45.1 as recipients and co-injecting whole bone marrow from CD45.1 littermates. To isolate the zsGreen-expressing cells, we kept the primary transplanted animals on doxycycline food for 2 days before bone marrow extraction. The recipient animals were similarly kept on doxycycline food to maintain shRNA expression. Due to the extremely low numbers of HSCs in each animal and the fact that the zsGreen^+^ cells are only a subset of the donor compartment, we sorted zsGreen^+^ LSK (Lineage^−^ Sca1^+^ cKit^+^), which contains a number of multipotent progenitors, in addition to HSCs (Figure 3D).

We transplanted 3,000 zsGreen-expressing LSK cells into each animal, together with a competitive dose of CD45.1 whole bone marrow. At week 3 post-reconstitution, we analyzed the peripheral blood for donor contributions resulting from negative control (*Renilla*), *Spehd*, or positive control (*Spi1*) knockdown. Whereas the contribution from the *Renilla*-knockdown cells reached 25% of nucleated peripheral blood, the ability of LSK cells to reconstitute the animals when depleted of *Spehd* was reduced to ∼10% on average, and as little as 5% in some animals (Figures 3E and 3F). Although all of the cells in the donor compartment should be zsGreen^+^, we saw a fraction of the donor being zsGreen^−^ (presumably due to silencing of the integrated cassette) (Figure S3D). This donor-derived zsGreen^−^ fraction is especially prominent in cells carrying *Spehd* shRNA 20 or *Spi1* shRNA 1, when the cells that managed to silence the transgene could have the greatest advantage (Figure S3D).

The effects are also seen if we look within each lineage for monocyte-macrophages and granulocytes and are apparent already at 3 weeks post-transplant (when the donor cells have not yet contributed to the B compartment) (Figures S3B and S3D). These data support an essential role for *Spehd* during HSC and/or progenitor cell self-renewal or differentiation that leads to impaired hematopoiesis when the lncRNA is depleted. Because we transplanted LSK, which encompasses both HSCs and multipotent progenitors, we are unable to identify the specific cell type affected to cause this depletion. The zsGreen contribution at later time points (up to week 9) was very low, even for the *Renilla* sh control, indicating a very small proportion of long-term reconstituting HSCs within the cell population transplanted into these animals. This, again, precludes us from distinguishing defects in the stem versus the progenitor compartment.

### The CMP Compartment Shows Defects in Respiratory Pathways when *Spehd* Is Depleted

**Figure 4:**
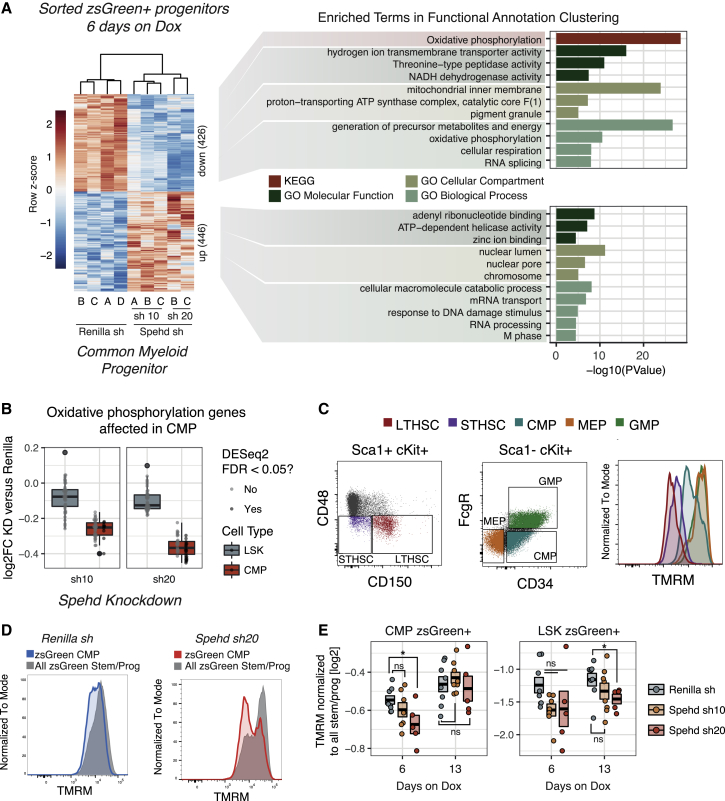
Depletion of *lnc12928* Results in Deficient Mitochondrial Function in Myeloid Progenitors (A) Heatmap of all of the differentially expressed genes (FDR < 0.05, DESeq) between *Spehd* and Renilla knockdown in CMP (left) and the results of functional annotation analysis (right). (B) Fold change for each shRNA against the lncRNA relative to *Renilla* in CMP and LSK for the genes in the oxidative phosphorylation pathway (KEGG) differentially expressed in CMP. Box and whiskers plots show the distribution of all of the genes represented. Boxplots correspond to the median and the 25^th^ and 75^th^ percentiles. The whiskers extend to the largest values, but no further than 1.5, the interquartile range (distance between the first and third quartiles), in which case the outliers are shown. (C) Representative flow cytometry plots showing the gating strategy for long-term (LT) and short-term (ST) repopulating HSCs (out of Lin^−^ Sca1^+^ cKit^+^) and CMPs, GMPs, and MEPs (out of Lin^−^ Sca1^−^ cKit^+^), and the corresponding distribution of the TMRM intensity for each population. (D) Representative flow cytometry plots of the TMRM of CMP and the TMRM in all of the progenitors and stem cells for Renilla shRNA and shRNA 20 against *lnc12928*. (E) TMRM (geometric mean) in CMP or LSK relative to overall TMRM in the sample (in all of the progenitors and stem cells) per animal. The box represents average and SEM. ^∗^p < 0.05; Mann-Whitney test. See also Figure S4.

To investigate the earliest consequence of *Spehd* knockdown and the cell types that are most affected by its depletion, we isolated cells from animals that had been reconstituted with shRNA-inducible HSCs and administered doxycycline for 6 days. When we examined genes that were differentially expressed upon lncRNA knockdown versus control CMPs, we identified 426 genes that were consistently downregulated upon suppression of the lncRNA (false discovery rate [FDR] <0.05). Those genes were strongly enriched for components of the oxidative phosphorylation pathway (Figure 4A). The signature is driven by 43 genes (Figure S4A) that are downregulated in CMPs (lncRNA knockdown versus control), but to a much lesser extent or not consistently affected in LSK (a stem and progenitor compartment that includes the HSCs) (Figures 4B and S4B). While the pathways enriched among the upregulated genes (Figure 4A) warrant further investigation, we chose to focus on the oxidative phosphorylation signature, given that 33% of the genes annotated for this pathway in the Kyoto Encyclopedia of Genes and Genomes (KEGG) were downregulated (43/130 genes annotated in the pathway).

HSCs are reported to primarily use glycolysis (Takubo et al., 2013) and have a lower respiratory capacity than multipotent or committed progenitors (de Almeida et al., 2017). Moreover, several mutants with mitochondrial and respiration defects have profound hematopoietic defects (Maryanovich et al., 2015, Yu et al., 2013), and HSCs with lower mitochondrial activity have a greater reconstitution capacity (Vannini et al., 2016). Therefore, it seemed possible that cells with a reduced expression of genes encoding respiratory chain components could encounter a roadblock when their metabolic requirements increase during their commitment to differentiation and expansion.

We therefore asked whether the 43 oxidative phosphorylation-pathway genes affected in CMP following *Spehd* depletion showed an increase in expression during the differentiation between LSK and CMP. In the *Renilla* control, all of the genes show, on average, increased expression, and 29 of the 43 are significantly upregulated (FDR <0.05). Consistent with our previous analysis, these genes fail to become induced in the CMPs depleted of lncRNA *Spehd* (Figure S4B). These results indicate a defect in the CMP population, which fails to activate the oxidative phosphorylation pathway genes and could partly explain the substantial reduction in differentiated cell output that we observed in this lncRNA knockdown.

*Spehd* was primarily cytoplasmic in AML cells as measured both by RT-qPCR following subcellular fractionation (Figure S4C) and single-molecule fluorescence *in situ* hybridization (FISH) (Figures S4D and S4E). We further validated the specificity of the single-molecule FISH by analyzing the number of molecules upon *Spehd* knockdown (Figures S4F and S4H). While the exact mechanism by which *Spehd* exerts its function remains to be elucidated, its localization would seem to exclude direct effects at the transcriptional level. This fits with the observation that the oxidative phosphorylation genes affected include three mitochondrially transcribed genes (Figure S4A, gene names in bold). Taking advantage of the RNA-seq data produced, we also examined the potential effects on the genes neighboring *lnc12928/Spehd*. For the well-known hematopoietic regulator *Gata2*, we observed a slight (on average, 15%) reduction, with both shRNAs against *Spehd* in CMPs (data not shown). While we cannot exclude that this plays a role in the phenotype, further experiments would be required to address whether such a modest change could have the impact that we observe.

To further investigate the deficiencies observed in the oxidative phosphorylation pathway, we transplanted animals following the same experimental design used for transcriptomic profiling (Figure 4A). We tested whether mitochondria function was affected by lncRNA knockdown in the common myeloid progenitor using tetramethylrhodamine methyl ester (TMRM). This cell-permeable dye is sequestered in active mitochondria and gives a progressively higher readout as the cells progress down the hematopoietic lineage to more committed states (Vannini et al., 2016) (Figure 4C). When we measured the relative TMRM levels for CMPs (see STAR Methods), we saw, on average, a reduction in the TMRM signal for both shRNAs targeting *Spehd* after doxycycline administration for 6 days (Figures 4D and 4E). While both shRNAs show the same trend, shRNA 20 provokes once again a stronger phenotype and is the only one to reach statistical significance. We note that TMRM accumulation could also correlate with bulk mitochondrial mass rather than their metabolic capacity. Animals analyzed after 13 days on doxycycline showed a depletion of relative TMRM levels in the LSK compartment (Figure 4E). This could be a downstream effect from a deficiency in CMPs, or it is possible that the LSK compartment would eventually show the same transcriptional deficiency in oxidative phosphorylation at later time points. No significant changes are observed in the megakaryocyte-erythroid progenitor (MEP) or GMP populations (Figure S4H). Determining the bone marrow cell types being depleted or blocked in their differentiation potential will be essential to fully understand the function of this lncRNA.

Our working hypothesis is that the ability of these defective progenitors to differentiate is reduced, leading to the strong phenotype we consistently observed. Follow-up studies will be required to determine whether these cell types are being depleted themselves or accumulate in the bone marrow. In addition, B cells were also greatly reduced when this lncRNA was knocked down (Figure S3C), and we suspect that a similar phenotype could be occurring in the lymphoid compartment at an unexplored time point, although an alternative mechanism that explains the lymphoid deficiency could also be contemplated.

## Discussion

We have previously cataloged lncRNA expression during mouse hematopoiesis and carried out a functional analysis of lncRNAs in the hematopoietic compartment in mouse leukemias as a proof of concept (Delás et al., 2017). Here, we have further explored lncRNA function in hematopoiesis, developing a strategy that allowed us to identify lncRNAs that play a role in HSC self-renewal or differentiation. Studies of HSCs pose many challenges because of their very low abundance and the lack of culture conditions that are suitable for their expansion. This prompted us to attempt to examine lncRNA function using *in vivo* HSC reconstitutions. While it is a powerful approach, the number of candidates that can be assessed using such assays is much lower than one can survey *in vitro*.

The strategy that we developed for candidate selection narrowed our study to five lncRNAs and was aimed at identifying non-coding species that could regulate the first steps of HSC commitment into myeloid or lymphoid lineages. However, this combination of differential expression, synteny, and conserved expression is broadly applicable to either other cell types in the hematopoietic lineage or other tissues.

Within the final candidate list, we noted that many of the genes surrounding the lncRNAs of interest had known roles in hematopoiesis (e.g., *Cdk6*, *Gata2*). Although a conserved role in *cis* cannot be excluded, we suspect that these coding and non-coding genes are simply regulated by the same tissue-specific elements in both mice and humans, which allows them to fulfill criteria that we set for candidate selection. One major drawback of our selection strategy was that we could have missed a functionally conserved lncRNA that does not show synteny between mice and humans.

*Spehd* emerged from this approach as an lncRNA required for stem cell differentiation. HSCs with reduced levels of *Spehd* show a decreased capacity for multilineage differentiation over time via either an HSC defect or a broad effect on all progenitors. Further transcriptomic analysis showed that this defect could, in part, be due to deficient oxidative phosphorylation in the CMP population. Even a slight defect in this pathway has the potential to be detrimental during a process in which cells exit a quiescent state and must undergo a rapid expansion, giving rise to a myriad of differentiated cell types. A recent report has identified the lncRNA here annotated as *lnc6689* (*2810468N07Rik*) also as being a regulator of oxidative phosphorylation in a microRNA-dependent manner (Sirey et al., 2018). Of course, the major questions that remain are a challenge in lncRNA biology more broadly—by what mechanism precisely does this non-coding RNA regulate the differentiation potential and the metabolic capacity of HSCs.

## STAR★Methods

### Key Resources Table

**Table:** 

REAGENT or RESOURCE	SOURCE	IDENTIFIER
**Antibodies**		

Mouse Lineage depletion kit	Miltenyi Biotec	Cat#130-090-858
Mouse BD Fc Block	BD Biosciences	Cat#553142; clone: 2.4G2
EPCR-PE	Stem Cell Technologies	Cat#60038PE; clone: MEPCR1560 (1560), PE
CD45-APC	eBioscience	Cat#17-0451-82; clone: 30-F11
CD150-PE/Cy7	BioLegend	Cat#115914; clone: TC15-12F12.2
CD48-FITC	BioLegend	Cat#103404; clone: HM48-1
CD45R/B220-APC	BioLegend	Cat#103212; clone: RA3-6B2
CD3-AF700	BioLegend	Cat#100216; clone: 17A2
CD11b (Mac1)-APC-Cy7	BioLegend	Cat#101226; clone: M1/70
Ly6G-APC-Cy7	BioLegend	Cat#127624; clone: 1A8
CD45.1-PE	BioLegend	Cat#110707; clone: A20
CD45.2-BV421	BioLegend	Cat#109832; clone: 104
CD45.1-AlexaFluor700	BioLegend	Cat#110724; clone: A20
Ly-6G-PE	BioLegend	Cat#127607; clone: 1A8
CD11b (Mac-1)-PE-Cy7	BioLegend	Cat#101216; clone: M1/70
CD34-Biotin	Invitrogen	Cat#13-0341-82; clone: RAM34
Streptavidin-PE	BioLegend	Cat#405203
CD45.2-V500	BD Biosciences	Cat#562130; clone: 104
CD45.2-BV510	BioLegend	Cat#109837; clone:104
CD117(cKit)-APC	eBioscience	Cat#17-1171-82; clone: 2B8
CD135 (Flt3)-BV421	BioLegend	Cat#135313; clone: A2F10
CD127 (IL7Rα)-PE-Cy7	BioLegend	Cat#135014; clone: A7R34
Ly-6A/E (Sca1)-BV605	BioLegend	Cat#108133; clone: D7
Ly-6A/E (Sca-1)-PerCPCy5.5	BioLegend	Cat#108124; clone: D7
CD34-AF700	Invitrogen	Cat#4324661; clone: RAM34
CD16/32 (FcγR)-BUV395	BD Biosciences	Cat#740217; clone: 2.4G2
CD48-BV421	BioLegend	Cat#103427; clone: HM48-1

**Bacterial and Virus Strains**		

Stellar Competent Cells	Clontech	Cat# 636766
Endura Electrocompetent Cells (DUOs)	Cambridge Bioscience	Cat# 60242-2

**Chemicals, Peptides, and Recombinant Proteins**		

LIVE/DEAD Fixable Violet Dead Cell Stain Kit	Thermo Scientific	Cat#L34963
Doxycycline-containing food (625 mg/kg)	Envigo	Cat# TD.01306
Doxycycline	Clontech	Cat# 631311
Ammonium Chloride Solution	Stem Cell Technologies	Cat# 07850
Tetramethylrhodamine Methyl Ester (TMRM)	Thermo Fisher Scientific	Cat# T668
BIT 9500	StemCell Technologies	Cat# 09500
β–mercaptoethanol	ThermoFisher Scientific	Cat# 21985023
Recombinant Mouse IL-11 Protein	R&D Systems	Cat# 418-ML-005
Recombinant Mouse SCF Protein	R&D Systems	Cat# 455-MC-050
ACK Red Blood Cell Lysis Buffer	Thermo Fisher Scientific	Cat# A1049201
Verapamil hydrochloride	Sigma	Cat# V4629-1G; CAS: 152-11-4
Trizol LS	ThermoFisher Scientific	Cat# 10296010
Chloroform	SIGMA-ALDRICH	Cat# 288306; CAS: 67-66-3
NucBlue® Fixed Cell ReadyProbes® Reagent	ThermoFisher Scientific	Cat# R37606
Fixable Viability Dye eFluor 780	eBioscience	Cat#65-0865-14
ProLong Diamond Antifade Mountant	ThermoFisher Scientific	Cat# P36961

**Critical Commercial Assays**		

SMART-Seq® v4 Ultra® Low Input RNA Kit for Sequencing	Takara Bio	Cat# 634890
Low Input Library Prep Kit	Takara Bio	Cat# 634947
NucleoSpin RNA XS	Machery Nagel	Cat# 740902.250
SMARTer RACE 5′/3′ Kit,	Clontech Laboratories, lnc	Cat# 634858

**Deposited Data**		

RNA sequencing data	This data	GEO: GSE124302

**Experimental Models: Cell Lines**		

MLL-AF9;NRAS^G12D^ AML	Lowe Laboratory (Zuber et al., 2011)	N/A
A20 cells	ATCC	RRID:CVCL_1940
293FT cells	Thermo Fisher Scientific	Cat# R70007

**Experimental Models: Organisms/Strains**		

C57BL/6-CD45.1; *Kit*^*W-41*^	David Kent, University of Cambridge	N/A
C57BL/6-CD45.1	Charles River (Kent, England)	N/A
C57BL6J	Charles River (Kent, England)	N/A

**Oligonucleotides**		

shRNAs	This study	Table S2
qPCR primers	This study	Table S2
smFISH probes	This study	Table S2
Primers for 3′/5′ RACE	This study	Table S2

**Recombinant DNA**		

ZIP-Neo (SFFV promoter)	Delás et al., 2017	N/A
T3G-zsGreen-ultramiR-SFFV-rtTA (L3zUSR)	This paper	N/A

**Software and Algorithms**		

shERWOOD	Knott et al., 2014	N/A
DESeq2	Love et al., 2014	N/A
UCSC’s liftover tool	UCSC	N/A
BEDTools Intersect	Quinlan laboratory at the University of Utah	https://bedtools.readthedocs.io/en/latest
STAR aligner	Dobin et al., 2013	N/A
htseq-count	Anders et al., 2015	N/A
IDT PrimerQuest tool	IDT	N/A
Picard	Broad Institute	http://broadinstitute.github.io/picard
DAVID 6.7	Huang et al., 2009	N/A
FlowJo Software	FlowJo, LLC	RRID:SCR_008520

### Contact for Reagent and Resource Sharing

Further information and requests for resources and reagents should be directed to and will be fulfilled by the Lead Contact, M. Joaquina Delás (joaquina.delas@crick.ac.uk).

### Experimental Model and Subject Details

#### Mice

Bone marrow transplantations of modified HSCs and subsequent analysis of peripheral blood and bone marrow from these mice were performed at the Cancer Research UK Cambridge Institute (Cambridge, UK). Female C57BL6J and C57BL/6-CD45.1 were purchased from Charles River (Kent, England) and used for tissue extraction (donor; C57BL6J) or as bone marrow transplant recipient (C57BL/6-CD45.1) at 9–12 weeks old. C57BL/6-CD45.1 whole bone marrow used in competitive transplants was obtained from female littermates to the recipient animals. C57BL/6-CD45.1; cKit-w41 animals were re-derived from embryos provided by David Kent at the Cambridge Institute for Medical Research. Both female and male were used as recipients in inducible transplantations at 9–12 weeks old. Induction of shRNAs was performed by administering doxycycline-containing food (625 mg/kg) for the days indicated at 10 weeks (Figure 3B and Figure 4A) or 12-13 weeks (Figure 3C, Figure 4D-E) post-transplant. These animal procedures were conducted in accordance with project and personal licenses issued under the United Kingdom Animals (Scientific Procedures) Act, 1986.

#### Cell lines

MLL-AF9;NRAS^G12D^ AML cells were obtained from the Lowe laboratory (Zuber et al., 2011) and cultured in RPMI-1640 with GlutaMax (GIBCO), supplemented with 10% heat-inactivated FBS (GIBCO) and 1% Penicillin/Streptomycin (GIBCO) under 7.5% CO_2_ culture conditions. This leukemia model was generated from fetal liver cells and the cell line established from it, also known as RN2, is of unspecified sex. A20 cells (sex unspecified, purchased from ATCC) were cultured in RPMI-1640 ATCC modification (GIBCO), supplemented with 10% heat-inactivated FBS (GIBCO), 1% Penicillin/Streptomycin (GIBCO), and 0.05 mM β–mercaptoethanol (GIBCO). 293FT cells (purchased from Thermo Fisher Scientific) were cultured as per manufacturer’s instructions. All cell lines tested negative for mycoplasma contamination by RNA-capture ELISA.

### Method Details

#### lncRNA candidate selection

The mouse RNaseq libraries from progenitors and differentiated cell types were previously published (Delás et al., 2017). Differential expression among hematopoietic progenitors was analyzed using DESeq2 (Love et al., 2014) and lncRNAs were considered to be differentially expressed if the fold change exceeded 2 and FDR < 0.05 in the comparisons indicated in the main text. Synteny was determined by converting the genomic interval for the mouse lncRNAs of interest from the mm10 to hg38 assembly using the UCSC’s liftover tool. The presence of human lncRNAs in these regions was verified using “BEDTools Intersect” with the GENCODE v22 lncRNA annotation. Human RNaseq expression data from cord progenitor types was obtained from previously existing datasets (Chen et al., 2014). Reads were mapped with the STAR aligner (Dobin et al., 2013) against the hg38 assembly, and fragment counting was performed with htseq-count (Anders et al., 2015). To compute expression correlation between mouse and human we first selected lncRNAs with an average expression across the human cell types of more than 20 normalized counts (value determined from the empirical count distributions). DESeq2 was used to calculate variance-stabilized data for each cell type in human and mouse. Expression correlation was computed between the median values for HSC, CMP, GMP and CLP in mouse versus human using Pearson correlation.

#### shRNAs design and cloning

shRNAs were predicted using the shERWOOD computation algorithm (Knott et al., 2014) as previously described for lncRNAs (Delás et al., 2017). shRNAs were cloned into the appropriate vectors, with ultramiR backbone: ZIP-Neo (constitutive bone marrow transplantations), or T3G-zsGreen-ultramiR-SFFV-rtTA (L3zUSR) (clonal inducible cell lines, inducible bone marrow transplantations) as previously described (Knott et al., 2014).

#### Virus production

In brief, virus was prepared in 15 cm dishes using 293FT cells (Thermo Fisher Scientific). The transfection mixture contained 32 μg of DNA vector, 12.5 μg of pMDL, 6.25 μg of CMV-Rev, 9 μg of VSV-G, 200 μg of Pasha siRNA (QIAGEN Custom siRNA CGGGTGGATCATGACATTCCA, QIAGEN), 125 μl 2.5M of CaCl_2_ brought to 1250 μl with H_2_O and bubbled into 1250 μl 2X HBS. Media was changed to IMDM supplemented with 10% heat-inactivated FBS right before transfection and collected in 16 mL of the same media. 38 mL of viral supernatant was ultracentrifuged for 2.5 hours at 25,000 rpm at 4°C, and resuspended in 100 μl of D-PBS (GIBCO). Viral titer was determined by infection of 293FT cells (Thermo Fisher Scientific) at various viral dilutions and percent infection was measured by flow cytometry analysis of the fluorescent protein expressed.

#### HSC transplantation

Bone marrow from C57BL/6 mice was extracted by flushing, filtered through a 0.30 μm filter and lineage depleted (Mouse Lineage depletion kit, Miltenyi Biotec 130-090-858). Cells were stained with EPCR-PE, CD45-APC, CD150-PE/Cy7 and CD48-FITC. DAPI or LIVE/DEAD Fixable Violet Dead Cell Stain Kit (Thermo Scientific L34963) was used for dead cell exclusion. Sorting of highly pure E-SLAM HSCs (on a FACSAria IIU, BD Biosciences) and short-term (∼20 hours) culture was performed as previously described (Kent et al., 2009). In short, 1000 live EPCR^+^CD45^+^CD150^+^CD48^–^ lineage negative cells were sorted in 100 μl of media: Iscove modified Dulbecco medium supplemented with 100 U/mL penicillin, 100 μg/mL streptomycin, 1X BIT (bovine serum albumin, insulin, transferring; purchased as 5X BIT StemCell Technologies), and 10^−4^ M β–mercaptoethanol (GIBCO) plus 20 ng/mL interleukin-11 (IL-11; R&D Systems) and 300 ng/mL Steel factor (R&D Systems)). Ultracentrifuged viral supernatant was added aiming for a final concentration of ∼2 x 10^7^ IU/ml following the sorting. Each well was used to inject four animals; cells were washed prior to injecting to remove remaining viral particles. Transplants with the constitutive vector (Figure 2) were performed into lethally irradiated (900 cGy in two split doses) C57BL/6-CD45.1 animals co-injecting 2 x 10^5^ nucleated whole bone marrow cells (from C57BL/6-CD45.1 animals) per animal. Transplants with the inducible vector (Figures 3A–3C and 4) were performed into sub-lethally irradiated (450 cGy) C57BL/6-CD45.1; cKit-w41 animals without competitive cells.

#### LSK re-transplantation

Re-transplants (Figures 3C–3F) were performed following the constitutive vector protocol (into C57BL/6-CD45.1with competitive dose) using Lin^–^Sca1^+^cKit^+^ (LSK) zsGreen^+^ cells isolated from transplanted animals. In the case of the re-transplants (Figures 3E–3F), the cells were re-injected the same day they were extracted.

#### Peripheral blood analysis – constitute shRNA vector

Blood from transplants with the constitutive vector was analyzed starting from 4 weeks after transplantation and every 4 weeks thereafter. 50–75 μl of blood was extracted from the animals’ tail vein into heparin coated capillary tubes. Red blood cell lysis was performed using Ammonium Chloride Solution (Stem Cell Technologies). Samples were then stained with B220-APC, CD3-AF700, CD11b (Mac1)-APC-Cy7, Ly6G-APC-Cy7, CD45.1-PE, and CD45.2-BV421, and analysis was done on an LSR Fortessa (BD Biosciences). Flow data analysis was performed using FlowJo and statistical analysis using R. All animals used for data analysis were required to have > 20% donor-derived cells at 16-weeks post-transplant.

#### Peripheral blood analysis – inducible shRNA vector

For blood analysis from the inducible vector transplants, blood was stained with CD45.1-AlexaFluor700, CD45.2-BV421, CD45R/B220-APC, Ly-6G-PE, CD11b (Mac-1)-PE-Cy7 and Fixable Viability Dye eFluor 780 (eBioscience) for dead cell exclusion, and the data acquired using a LSR Fortessa (BD Biosciences). Animals were analyzed from 2 days after doxycycline administration, as indicated in the Figures 3B and 3C. Flow data analysis was performed using FlowJo and plotting and statistical analysis using R.

#### Peripheral blood analysis – re-transplantations

For re-transplanted animals, blood was stained as for the inducible shRNA vector-transplanted animals, and analyzed at 3 weeks post re-transplant, as indicated in Figure 3F. Data was acquired using a LSR Fortessa (BD Biosciences) and FlowJo and R were used for data analysis.

#### Bone marrow cell isolation and analysis

Bone marrow was isolated from the femurs of euthanized animals at various time points following transplantation. Whole bone marrow was extracted by flushing and was filtered through a 0.3 μm filter. To obtain progenitor populations, 5 × 10^7^ bone marrow cells were lineage depleted (Mouse Lineage depletion kit, Miltenyi Biotec 130-090-858). Cells were stained with FcγR-BUV395, CD34-Biotin-Streptavidin-PE, CD45.1-AF700, CD45.2-V500 or BV510, cKit-APC, Flt3-BV421, IL7Rα-PE-Cy7, Sca1-BV605. Fixable Viability Dye eFluor780 (eBioscience) was used for dead cell exclusion, and analysis was performed on a FACSAria IIU (BD Biosciences). For analysis of differentiated populations, red blood cell lysis was performed on whole bone marrow using ACK Lysing Buffer (Thermo Fisher Scientific). Remaining cells were then stained with B220-APC, CD3-AF700, CD11b (Mac1)-APC-Cy7, Ly6G-APC-Cy7, CD45.1-PE, and CD45.2-BV421, and analysis was done on a FACSAria IIU (BD Biosciences). Flow data analysis was performed using FlowJo and plotting and statistical analysis using R.

#### TMRM staining and analysis

For Tetramethylrhodamine, Methyl Ester, Perchlorate (TMRM, Thermo Fisher Scientific, Cat no: T668) analysis, freshly isolated bone marrow was incubated in TMRM (20 nM) and Verapamil (50 μM) for exactly 30 min at 37°C, then washed and depleted of differentiated cell types as described above. Lineage depleted cells were stained with Ly-6A/E(Sca-1)-PerCPCy5.5, CD117(cKit)-APC, CD34-AF700, CD16/32 (FcγR)-BUV395, CD48-BV421,CD150-PE-Cy7 and analyzed in a FACSARIA IIU (BD Bioscience). Flow data analysis was performed using FlowJo. Relative TMRM for each indicated population was calculated by normalizing the TMRM geometric mean intensity in that population of interest by the TMRM level of all the stem and progenitor cells – LSK and Lin^–^ Sca1^–^ cKit^+^ together, to control for inter animal differences in TMRM signal. Plotting and statistical analysis was performed using R.

#### lncRNA knockdown analysis

Knockdown measurements were performed after inducing shRNA expression for 48h in clonal cell lines selected for uniform zsGreen induction. We performed each experiment with at least 3 independent clonal cell lines for each shRNA. For all lncRNAs knockdown efficiency was assessed in MLL-AF9;NRAS^G12D^ AML cells (RN2) (Zuber et al., 2011), with the exception of lnc6689 which was assessed in A20 cells. RNA was extracted using the RNeasy Mini Kit (QIAGEN), including treatment with the DNase Set (QIAGEN). Reverse transcription was performed using Superscript III (ThermoFisher Scientific), with 4 μg of RNA and 1 μl of 50 μM oligo(dT)20. Primers were designed using IDT PrimerQuest tool or chosen from IDT’s pre-designed set when available. Fast SYBR Green (ThermoFisher Scientific) was used for qPCR. Primer pair efficiency was assessed using serial dilutions of cDNA from untreated RN2 or A20 cells, and melting curves were examined to ensure the presence of only one amplicon. *Gapdh* was used as a housekeeping normalization control in the delta-delta-Ct analysis.

#### Subcellular Fractionation

Subcellular fractionation of MLL-AF9;NRAS^G12D^ AML cells (RN2) was performed as previously published (Gagnon et al., 2014). In brief, 2 x 10^7^ cells were split in two equal aliquots, one for total RNA isolation and one for cellular fractionation. For fractionation, cells were washed in ice-cold 1X PBS and incubated on ice for 10 min in 380 μl of ice-cold Hypotonic Lysis Buffer, HLB, (10 mM Tris pH 7.5, 10 mM NaCl, 3 mM MgCl_2_, 0.3% Igepal CA-630 (SIGMA-ALDRICH) and 10% glycerol) supplemented with 100 U of SUPERase-In RNase Inhibitor (ThermoFisher Scientific). The sample was centrifuged at 1,000 g at 4°C for 3 min. The supernatant (cytoplasmic fraction) was mixed with 1 mL of RNA Precipitation Solution, RPS, (0.5 mL 3 M sodium acetate pH 5.5 and 9.5 mL ethanol) and stored at −20°C for at least 1 hour. The pellet (semi-pure nuclei) was washed three times with 1 mL of ice-cold HBL and resuspended in 1 mL of Trizol LS (ThermoFisher Scientific). The cytoplasmic fractions (in RPS) was vortexed for 30 s and then centrifuged at 18,000 g at 4°C for 15 min. The pellet was washed in ice-cold 70% (vol/vol) ethanol and resuspended in 1 mL of Trizol LS. RNA from either fraction or from the total sample (in Trizol) was extracted by adding 200 μl of Chloroform (SIGMA-ALDRICH), mixing and incubating at room temperature for 10 min. The phases were subsequently separated by centrifugation at 18,000 g at room temperature for 10 min.

An equal volume of 70% (vol/vol) ethanol was added to the aqueous phase and RNA was isolated using the RNeasy Mini Kit (QIAGEN), including DNase treatment (QIAGEN), performed according to manufacturer’s instructions. The RNA was eluted in 30 μl of Nuclease-free water. Reverse transcription was performed using SuperScript III (ThermoFisher Scientific) as described above but using equal volumes of RNA solution from each fraction sample (nuclear, cytoplasmic or total RNA). Two primer pairs were used for *lnc12928*, in addition to primers for *Malat1* as a nuclear control and *Gapdh* as a cytoplasmic control. Cytoplasmic enrichment for each primer pair was calculated as the 2^-(Cytoplasmic Ct – Total Ct)^. The nuclear enrichment was calculated accordingly. This does not provide an absolute measurement of the proportion of lncRNA in each fraction but rather allows one to compare the enrichment in each fraction to the known controls.

#### Single molecule RNA FISH

SmRNA FISH probes were ordered from *Stellaris* conjugated to Quasar® 570 Dye. *Malat1* and *Gapdh* mouse controls were selected from the ShipReady Stellaris probe sets. The protocol was performed according to manufacturer’s instructions for cells in suspension (Biosearch Technologies). 5 × 10^6^ cells were washed in 1 mL of 1X PBS and the pellet was fixed in 1 mL of Fixation Buffer (3.7% formaldehyde in 1X PBS) at room temperature for 10 min. The fixed cells were washed three times with 1X PBS and then permeabilized at 4°C for at least 1 hour using 70% (vol/vol) ethanol. 500 μl of fixed/permeabilized cells were washed in 500 μl of Wash Buffer A (10% formamide in 1X Stellaris Wash Buffer A) before incubating in 100 μl of Hybridization Buffer (10% formamide in Stellaris Hybridization Buffer) containing 125 nM probe at 37°C in the dark overnight. The sample was centrifuged to pellet the cells and 50% of the Hybridization Buffer was removed. The pellet was then washed in Wash Buffer A and incubated in the dark at 37°C for 30 min with 500 μl of this same buffer. The nuclei were stained with NucBlue Fixed Cell Stain (ThermoFisher Scientific), the cells were washed with Stellaris Wash Buffer B and seeded on a clean glass microscope slide in one drop of ProLong Diamond Antifade Mountant (ThermoFisher Scientific). The cells were imaged in a Nikon TE2000 Widefield inverted microscope. Z stacks were acquired by sampling every 0.3 μm. For subcellular localization analysis of the lnc12928 probe, the nuclear or cytoplasmic localization of the signal was established in the Z stacks where the smFISH was detected. The fraction of cytoplasmic or nuclear signal per cell was calculated.

#### 5′-/3′-RACE

5′-/3′-rapid amplification of cDNA ends (RACE) of MLL-AF9;NRAS^G12D^ AML cells (RN2) and lineage-depleted murine bone marrow progenitor populations (Mouse Lineage depletion kit, Miltenyi Biotec 130-090-858) was performed according to manufacturer’s instructions (SMARTer RACE 5′/3′ Kit, Clontech Laboratories, lnc 634858). 4 primers were designed for each 5′- and 3′-RACE PCR according to Section IV of the SMARTer RACE 5′/3′ Kit User Manual. All primers were used on AML cells, and subsequently the ones producing the longest insert were used in lineage-depleted primary cells. Total RNA was isolated using the RNeasy Mini Kit (QIAGEN), including DNase treatment (QIAGEN), and the RNA quality was assessed evaluating the RNA integrity number (RIN) (Agilent 2100 BioAnalyzer, Agilent Technologies), which were always above 9. RNA samples with an RNA integrity number less than 7 should be discarded. In brief, 1 μg of total RNA was converted into 5′-/3′-RACE-Ready first-strand cDNA using Universal 5′-/3′-CDS Primer A, SMARTScribe Reverse Transcriptase (RT) and, for the 5′-RACE cDNA synthesis reaction, the SMARTer II A Oligonucleotide as an extended template for the SMARTScribe RT. 2.5 μL of the resulted 5′-/3′-RACE-Ready cDNA samples were used to generate 5′ and 3′ cDNA fragments in PCR reactions combining them with SeqAmp DNA Polymerase and the gene-specific primers. The PCR reaction was performed running 30 cycles of PCR Program 2 described in Section VI of the SMARTer RACE 5′/3′ Kit User Manual. RACE products were then cloned into the linearized pRACE vector using the In-Fusion HD Cloning Kit included in the SMARTer RACE 5′/3′ Kit. In order to determine the presence of RACE insert of interest, the DNA was analyzed by restriction digest with EcoRI and HindIII which flank the cloning site. The clones containing the largest gene-specific inserts were sequenced with M13 primers. The quality of the sanger reads was analyzed manually. Subsequently, sequences were mapped to the mm10 genome with bwa. The 5′ end of the 3′-RACE products and the 3′ end of the 5′-RACE products only extended to the primer used in the reaction. All the lncRNA 5′ and 3′ ends are represented for each sample.

#### RNaseq of Spehd-depleted progenitors

LSK and CMP populations were sorted from the bone marrow isolated from transplanted mice as described for bone marrow analysis. RNaseq libraries were prepared as previously described (Delás et al., 2017). In brief, RNA was extracted using the NucleoSpin RNA XS Kit (Machery Nagel). cDNA and libraries were prepared using the SMART-Seq v4 Ultra Low Input RNA Kit for Sequencing (Clontech), SeqAmp DNA Polymerase (Clontech), and Low Input Library Prep Kit (Clontech). Samples were pooled and run on a HiSeq 4000.

#### Spehd-depleted progenitors RNaseq data processing

RNaseq libraries were mapped with STAR aligner (Dobin et al., 2013) against the mm10 mouse genome assembly using default parameters. Duplicate alignments were removed from the resulting BAM files with Picard (http://broadinstitute.github.io/picard). HTSeq-count (Anders et al., 2015) was used to calculate gene counts and subsequently input them into DESeq2 (Love et al., 2014) for quality control analysis, size normalization and variance dispersion corrections. For functional annotation analysis, DESeq2 was used to calculate differentially expressed genes (FDR < 0.05). Significantly downregulated or upregulated genes in CMPs upon lncRNA knockdown were used independently as input for DAVID 6.7 (Huang et al., 2009). The categories shown are the result of performing Functional Annotation clustering for each category (Gene Ontology Biological Process, GO Molecular Function, GO Cellular Compartment of KEGG). Terms with Bonferroni-corrected p value < 0.05 for the first 5 clusters are represented.

### Quantification and Statistical Analysis

Specific data analysis workflows, as well as parameters such as center, error bars and significance are described in the Results, Figure legends and STAR Methods sections. Plotting and statistical analyses were performed in R version 3.4.4 (2018-03-15). For RNaseq analysis, default parameters were used in all software unless otherwise specified. The version details were as follows: STAR aligner version 2.5.2a, Picard tools 1.131, ‘HTSeq’ framework, version 0.7.2. used with GENCODE vM11 transcriptome, DESeq2_1.16.1.

### Data and Software Availability

The accession number for the RNaseq from LSK and CMP data reported in this paper is GEO: GSE124302.

## Supplemental Information

Document S1. Figures S1–S4

Table S1. List of *In Vivo* lncRNAs with Human and Mouse Coordinates, Related to Figure 1

Table S2. Oligonucleotides, Related to STAR Methods

Document S2. Article plus Supplemental Information

## References

[bib1] Alvarez-Dominguez J.R., Hu W., Yuan B., Shi J., Park S.S., Gromatzky A.A., van Oudenaarden A., Lodish H.F. (2014). Global discovery of erythroid long noncoding RNAs reveals novel regulators of red cell maturation. Blood.

[bib2] Anders S., Pyl P.T., Huber W. (2015). HTSeq--a Python framework to work with high-throughput sequencing data. Bioinformatics.

[bib3] Cabezas-Wallscheid N., Klimmeck D., Hansson J., Lipka D.B., Reyes A., Wang Q., Weichenhan D., Lier A., von Paleske L., Renders S. (2014). Identification of regulatory networks in HSCs and their immediate progeny via integrated proteome, transcriptome, and DNA methylome analysis. Cell Stem Cell.

[bib4] Challen G.A., Sun D., Jeong M., Luo M., Jelinek J., Berg J.S., Bock C., Vasanthakumar A., Gu H., Xi Y. (2011). Dnmt3a is essential for hematopoietic stem cell differentiation. Nat. Genet..

[bib5] Challen G.A., Sun D., Mayle A., Jeong M., Luo M., Rodriguez B., Mallaney C., Celik H., Yang L., Xia Z. (2014). Dnmt3a and Dnmt3b have overlapping and distinct functions in hematopoietic stem cells. Cell Stem Cell.

[bib6] Chen L., Kostadima M., Martens J.H.A., Canu G., Garcia S.P., Turro E., Downes K., Macaulay I.C., Bielczyk-Maczynska E., Coe S., BRIDGE Consortium (2014). Transcriptional diversity during lineage commitment of human blood progenitors. Science.

[bib7] de Almeida M.J., Luchsinger L.L., Corrigan D.J., Williams L.J., Snoeck H.-W. (2017). Dye-Independent Methods Reveal Elevated Mitochondrial Mass in Hematopoietic Stem Cells. Cell Stem Cell.

[bib8] Delás M.J., Hannon G.J. (2017). lncRNAs in development and disease: from functions to mechanisms. Open Biol..

[bib9] Delás M.J., Sabin L.R., Dolzhenko E., Knott S.R., Munera Maravilla E., Jackson B.T., Wild S.A., Kovacevic T., Stork E.M., Zhou M. (2017). lncRNA requirements for mouse acute myeloid leukemia and normal differentiation. eLife.

[bib10] Dobin A., Davis C.A., Schlesinger F., Drenkow J., Zaleski C., Jha S., Batut P., Chaisson M., Gingeras T.R. (2013). STAR: ultrafast universal RNA-seq aligner. Bioinformatics.

[bib11] Fazi F., Racanicchi S., Zardo G., Starnes L.M., Mancini M., Travaglini L., Diverio D., Ammatuna E., Cimino G., Lo-Coco F. (2007). Epigenetic silencing of the myelopoiesis regulator microRNA-223 by the AML1/ETO oncoprotein. Cancer Cell.

[bib12] Fukao T., Fukuda Y., Kiga K., Sharif J., Hino K., Enomoto Y., Kawamura A., Nakamura K., Takeuchi T., Tanabe M. (2007). An evolutionarily conserved mechanism for microRNA-223 expression revealed by microRNA gene profiling. Cell.

[bib13] Gagnon K.T., Li L., Janowski B.A., Corey D.R. (2014). Analysis of nuclear RNA interference in human cells by subcellular fractionation and Argonaute loading. Nat. Protoc..

[bib14] Grass J.A., Jing H., Kim S.-I., Martowicz M.L., Pal S., Blobel G.A., Bresnick E.H. (2006). Distinct functions of dispersed GATA factor complexes at an endogenous gene locus. Mol. Cell. Biol..

[bib15] Hezroni H., Koppstein D., Schwartz M.G., Avrutin A., Bartel D.P., Ulitsky I. (2015). Principles of long noncoding RNA evolution derived from direct comparison of transcriptomes in 17 species. Cell Rep..

[bib16] Hu W., Yuan B., Flygare J., Lodish H.F. (2011). Long noncoding RNA-mediated anti-apoptotic activity in murine erythroid terminal differentiation. Genes Dev..

[bib17] Huang W., Sherman B.T., Lempicki R.A. (2009). Systematic and integrative analysis of large gene lists using DAVID bioinformatics resources. Nat. Protoc..

[bib18] Iwasaki H., Somoza C., Shigematsu H., Duprez E.A., Iwasaki-Arai J., Mizuno S., Arinobu Y., Geary K., Zhang P., Dayaram T. (2005). Distinctive and indispensable roles of PU.1 in maintenance of hematopoietic stem cells and their differentiation. Blood.

[bib19] Johnnidis J.B., Harris M.H., Wheeler R.T., Stehling-Sun S., Lam M.H., Kirak O., Brummelkamp T.R., Fleming M.D., Camargo F.D. (2008). Regulation of progenitor cell proliferation and granulocyte function by microRNA-223. Nature.

[bib20] Kent D.G., Copley M.R., Benz C., Wöhrer S., Dykstra B.J., Ma E., Cheyne J., Zhao Y., Bowie M.B., Zhao Y. (2009). Prospective isolation and molecular characterization of hematopoietic stem cells with durable self-renewal potential. Blood.

[bib21] Kerenyi M.A., Shao Z., Hsu Y.-J., Guo G., Luc S., O’Brien K., Fujiwara Y., Peng C., Nguyen M., Orkin S.H. (2013). Histone demethylase Lsd1 represses hematopoietic stem and progenitor cell signatures during blood cell maturation. eLife.

[bib22] Knott S.R.V., Maceli A., Erard N., Chang K., Marran K., Zhou X., Gordon A., Demerdash O.E., Wagenblast E., Kim S. (2014). A computational algorithm to predict shRNA potency. Mol. Cell.

[bib23] Lara-Astiaso D., Weiner A., Lorenzo-Vivas E., Zaretsky I., Jaitin D.A., David E., Keren-Shaul H., Mildner A., Winter D., Jung S. (2014). Immunogenetics. Chromatin state dynamics during blood formation. Science.

[bib24] Laurenti E., Frelin C., Xie S., Ferrari R., Dunant C.F., Zandi S., Neumann A., Plumb I., Doulatov S., Chen J. (2015). CDK6 levels regulate quiescence exit in human hematopoietic stem cells. Cell Stem Cell.

[bib25] Lechman E.R., Gentner B., van Galen P., Giustacchini A., Saini M., Boccalatte F.E., Hiramatsu H., Restuccia U., Bachi A., Voisin V. (2012). Attenuation of miR-126 activity expands HSC in vivo without exhaustion. Cell Stem Cell.

[bib26] Love M.I., Huber W., Anders S. (2014). Moderated estimation of fold change and dispersion for RNA-seq data with DESeq2. Genome Biol..

[bib27] Luo M., Jeong M., Sun D., Park H.J., Rodriguez B.A.T., Xia Z., Yang L., Zhang X., Sheng K., Darlington G.J. (2015). Long non-coding RNAs control hematopoietic stem cell function. Cell Stem Cell.

[bib28] Mangiavacchi A., Sorci M., Masciarelli S., Larivera S., Legnini I., Iosue I., Bozzoni I., Fazi F., Fatica A. (2016). The miR-223 host non-coding transcript linc-223 induces IRF4 expression in acute myeloid leukemia by acting as a competing endogenous RNA. Oncotarget.

[bib29] Maryanovich M., Zaltsman Y., Ruggiero A., Goldman A., Shachnai L., Zaidman S.L., Porat Z., Golan K., Lapidot T., Gross A. (2015). An MTCH2 pathway repressing mitochondria metabolism regulates haematopoietic stem cell fate. Nat. Commun..

[bib30] Melé M., Rinn J.L. (2016). “Cat’s Cradling” the 3D Genome by the Act of LncRNA Transcription. Mol. Cell.

[bib31] Orkin S.H., Zon L.I. (2008). Hematopoiesis: an evolving paradigm for stem cell biology. Cell.

[bib32] Paralkar V.R., Mishra T., Luan J., Yao Y., Kossenkov A.V., Anderson S.M., Dunagin M., Pimkin M., Gore M., Sun D. (2014). Lineage and species-specific long noncoding RNAs during erythro-megakaryocytic development. Blood.

[bib33] Schütte J., Wang H., Antoniou S., Jarratt A., Wilson N.K., Riepsaame J., Calero-Nieto F.J., Moignard V., Basilico S., Kinston S.J. (2016). An experimentally validated network of nine haematopoietic transcription factors reveals mechanisms of cell state stability. eLife.

[bib34] Shay T., Jojic V., Zuk O., Rothamel K., Puyraimond-Zemmour D., Feng T., Wakamatsu E., Benoist C., Koller D., Regev A., ImmGen Consortium (2013). Conservation and divergence in the transcriptional programs of the human and mouse immune systems. Proc. Natl. Acad. Sci..

[bib35] Sirey T.M., Roberts K., Haerty W., Bedoya-Reina O., Rogatti-Granados S., Tan J., Li N., Heather L., Cooper S., Marques A., Ponting C. (2018). Cerox1 and microRNA-488-3p noncoding RNAs jointly regulate mitochondrial complex I catalytic activity. BioRxiv.

[bib36] Snow J.W., Trowbridge J.J., Fujiwara T., Emambokus N.E., Grass J.A., Orkin S.H., Bresnick E.H. (2010). A single cis element maintains repression of the key developmental regulator Gata2. PLoS Genet..

[bib37] Takubo K., Nagamatsu G., Kobayashi C.I., Nakamura-Ishizu A., Kobayashi H., Ikeda E., Goda N., Rahimi Y., Johnson R.S., Soga T. (2013). Regulation of glycolysis by Pdk functions as a metabolic checkpoint for cell cycle quiescence in hematopoietic stem cells. Cell Stem Cell.

[bib38] Trowbridge J.J., Snow J.W., Kim J., Orkin S.H. (2009). DNA methyltransferase 1 is essential for and uniquely regulates hematopoietic stem and progenitor cells. Cell Stem Cell.

[bib39] Vannini N., Girotra M., Naveiras O., Nikitin G., Campos V., Giger S., Roch A., Auwerx J., Lutolf M.P. (2016). Specification of haematopoietic stem cell fate via modulation of mitochondrial activity. Nat. Commun..

[bib40] Wagner L.A., Christensen C.J., Dunn D.M., Spangrude G.J., Georgelas A., Kelley L., Esplin M.S., Weiss R.B., Gleich G.J. (2007). EGO, a novel, noncoding RNA gene, regulates eosinophil granule protein transcript expression. Blood.

[bib41] Yu W.-M., Liu X., Shen J., Jovanovic O., Pohl E.E., Gerson S.L., Finkel T., Broxmeyer H.E., Qu C.-K. (2013). Metabolic regulation by the mitochondrial phosphatase PTPMT1 is required for hematopoietic stem cell differentiation. Cell Stem Cell.

[bib42] Zandi S., Månsson R., Tsapogas P., Zetterblad J., Bryder D., Sigvardsson M. (2008). EBF1 is essential for B-lineage priming and establishment of a transcription factor network in common lymphoid progenitors. J. Immunol..

[bib43] Zhang X., Lian Z., Padden C., Gerstein M.B., Rozowsky J., Snyder M., Gingeras T.R., Kapranov P., Weissman S.M., Newburger P.E. (2009). A myelopoiesis-associated regulatory intergenic noncoding RNA transcript within the human HOXA cluster. Blood.

[bib44] Zuber J., McJunkin K., Fellmann C., Dow L.E., Taylor M.J., Hannon G.J., Lowe S.W. (2011). Toolkit for evaluating genes required for proliferation and survival using tetracycline-regulated RNAi. Nat. Biotechnol..

