# Supplementary material for: lncRNA Spehd Regulates Hematopoietic Stem and Progenitor Cells and Is Required for Multilineage Differentiation
Source: Cell Rep. 2019 Apr 16;27(3):719–729.e6. doi: 10.1016/j.celrep.2019.03.080 (PMC6484780; doi:10.1016/j.celrep.2019.03.080)
Supplement: Document S1. Figures S1–S4 [file mmc1.pdf]

Cell Reports, Volume *27*

Supplemental Information

lncRNA *Spehd* Regulates Hematopoietic Stem and Progenitor Cells and Is Required for Multilineage Differentiation

M. Joaquina Delás, Benjamin T. Jackson, Tatjana Kovacevic, Silvia Vangelisti, Ester Munera Maravilla, Sophia A. Wild, Eva Maria Stork, Nicolas Erard, Simon R.V. Knott, and Gregory J. Hannon

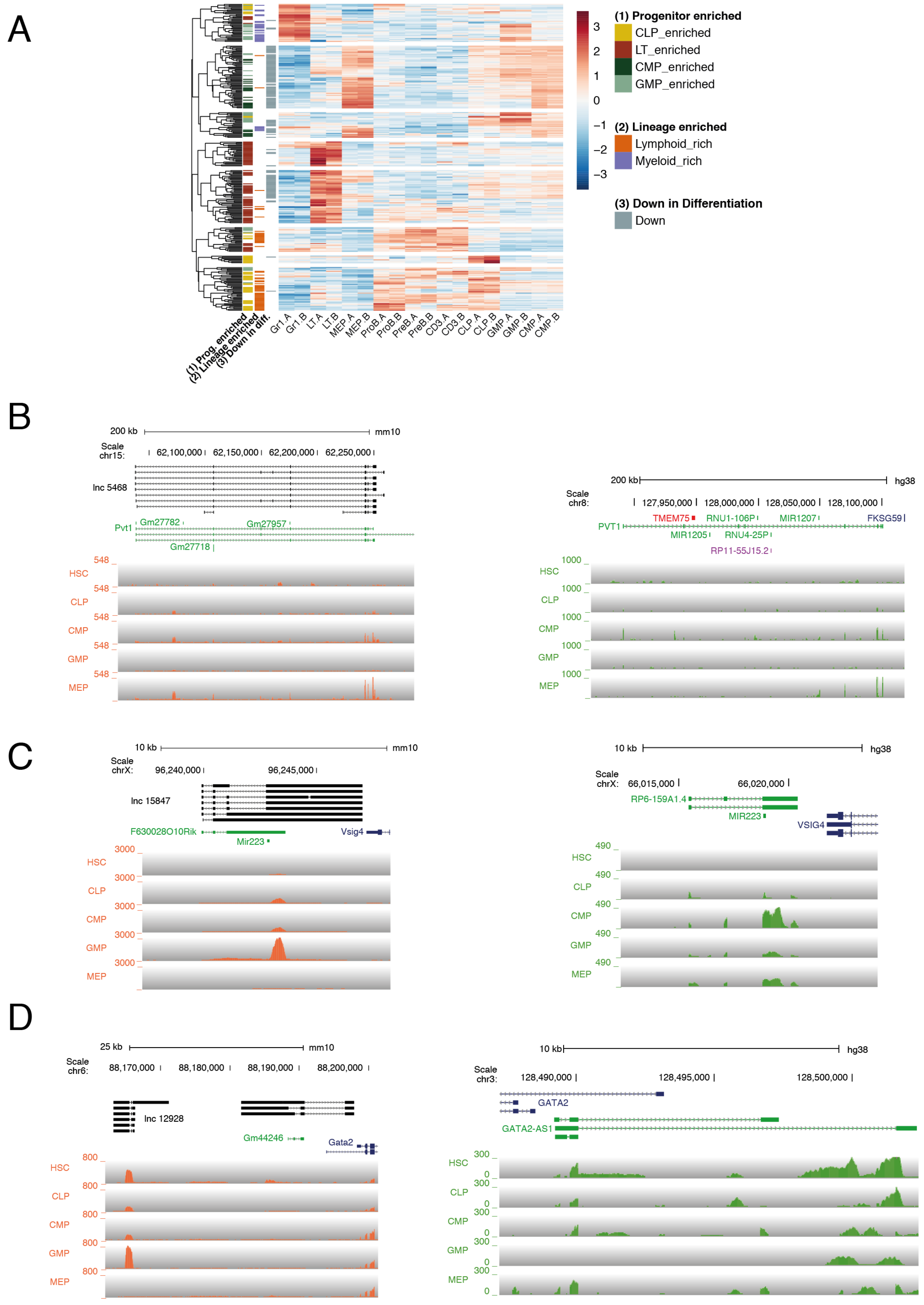

Figure S1. LncRNA candidate selection, Related to Figure 1.

(A) Heat map representing row-scaled expression for all the lncRNAs selected based on their expression patterns (Fig 1A, first panel)

(B-D) Genome browser plots of all the remaining 3 lncRNAs selected for *in vivo* studies not shown in Fig 1C-D.

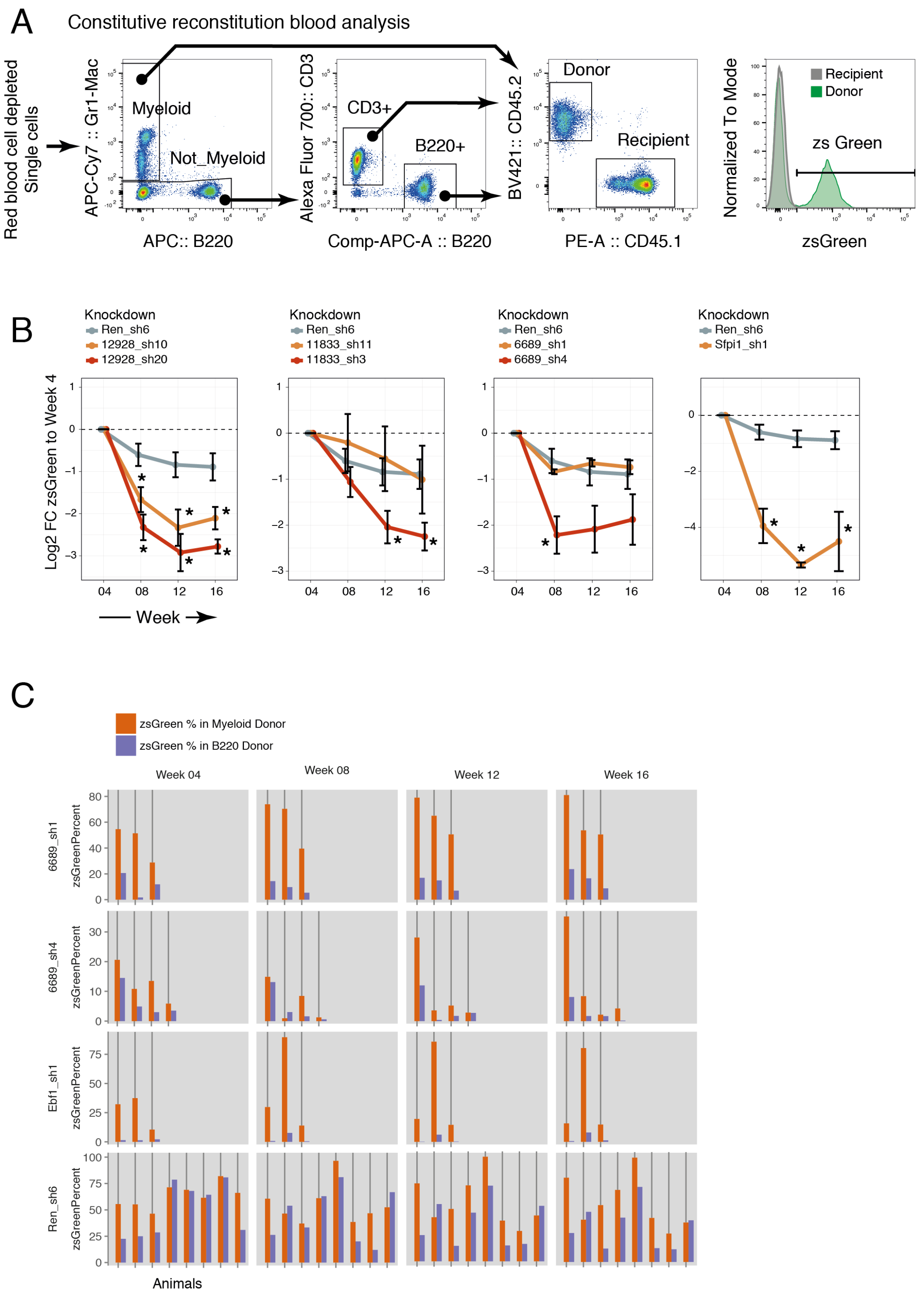

Figure S2. Peripheral blood analysis, Related to Figure 2.
(A) Summary of the flow cytometry analysis performed in the peripheral blood of animals transplanted with HSCs transduced with the constitutive vector.
(B) Relative ZsGreen % for control and lncRNA knockdown for all significantly-depleted conditions. Average across all animals is shown for each time point. Error bars represent s.e.m.
(C) zsGreen% within myeloid or B donor cells for the knockdowns indicated at the different time points. The animals are shown in the same order for the different time points.

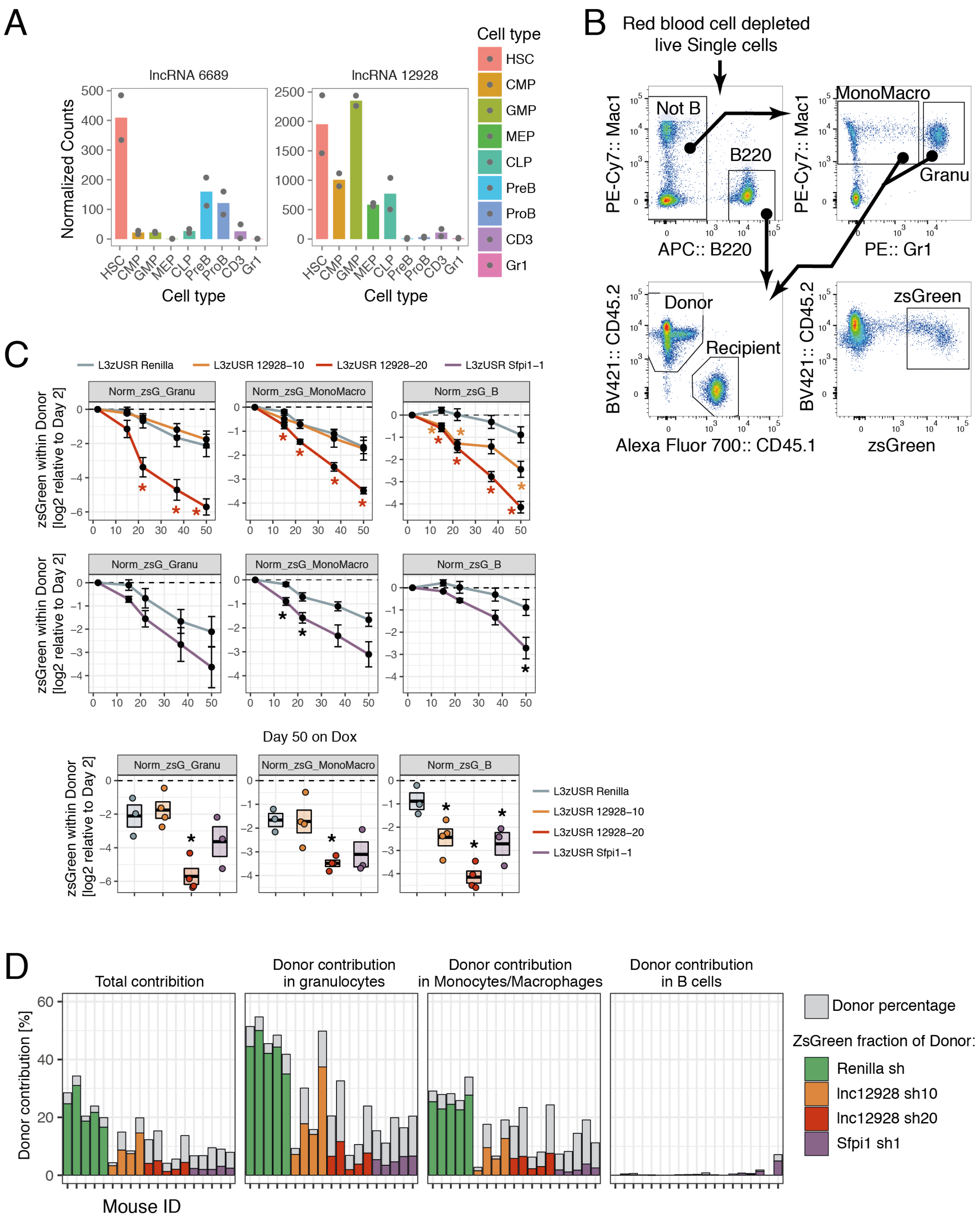

Figure S3. lncRNAs 12928 and 6689 expression and phenotype characterization, Related to Figure 3.
(A) Expression for lnc6689 and lnc12928 in all the normal blood cell types analyzed in our previous study. The height of the bar represents the average; the dots are the individual replicates.
(B) Summary of the flow cytometry analysis performed in the peripheral blood of animals transplanted with HSCs transduced with the inducible vector.
(C) Relative zsGreen% within each lineage for the conditions indicated (corresponds to Fig 3C). Black dots represent the average for all animals in each condition, error bars represent s.e.m. For the last timepoint, each animal is shown as a circle and the box represents average and s.e.m. (bottom panels).
(D) Bar graph representing the overall donor contribution (grey) and the zsGreen+ fraction within it (colored part) for each animal at 3 weeks post-transplant for overall blood or the lineages indicated.

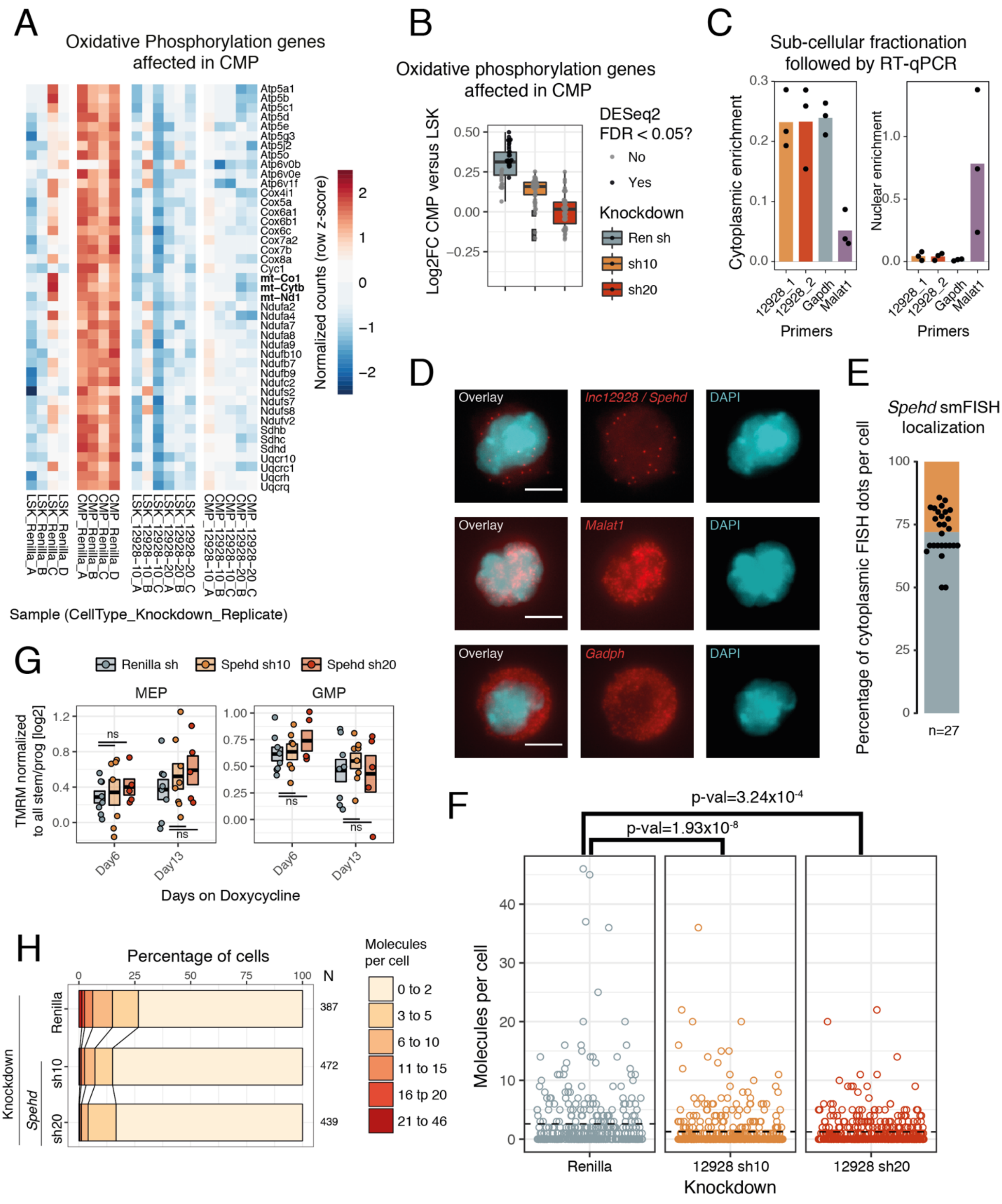

Figure S4. LncRNA *Spehd* affects oxidative phosphorylation and is predominantly cytoplasmic, Related to Figure 4.

(A) Heat map representing the row-scaled expression in the indicated samples for the 43 oxidative phosphorylation genes (KEGG) affected by lnc12928 knockdown in CMPs.

(B) Fold change in expression of the same genes in CMPs compared to LSK for each indicated short hairpin. Box and whiskers plots show distribution of all genes represented. Box plots correspond to the median, and the 25th and 75th percentiles. The whiskers extend to the largest values but no further than 1.5 the inter-quartile range (distance between the first and third quartiles), in which case the outliers are shown.

(C) Cytoplasmic and nuclear enrichment (see Experimental Procedures) for *lnc12928/Spehd*, *Malat1* (nuclear control) or *Gapdh* (cytoplasmic control). 12928_1 and 12928_2 correspond to the two primer pairs for lnc12928.

(D) Representative single molecule FISH (smFISH) images for *lnc12928/Spehd*, *Malat1* (nuclear control) or *Gapdh* (cytoplasmic control). Maximum intensity projections shown. Scale bar 5 μm.
(E) Quantification of the smFISH spots localization. Each dot represents one cells, its height being the percentage of *lnc12928/Spehd* single molecules observed in the cytoplasmic fraction. Height of the grey bar indicates overall average. Cytoplasmic/nuclear localization was determined based on their co-localization with DAPI signal in the stack where the signal was observed.
(F) Molecules per cell detected for each cell in the different conditions. Each circle represents a cell. The dash line represents the average number of molecules per condition. P-value indicated, Mann-Whitney test.
(G) TMRM (Geometric mean) in MEP or GMP relative to overall TMRM in the sample (in all progenitors and stem cells) per animal. Box represents average and s.e.m.
(H) Summary of smFISH quantification from (F) for control or *lnc12928/Spehd* knockdown. N, number of cells analyzed per condition.
